# Complete Transperitoneal Laparoscopic Nephroureterectomy in Circumcaval Ureter with Upper Tract TCC: Initial Case Report

**DOI:** 10.1089/cren.2015.29013.jsc

**Published:** 2015-10-01

**Authors:** Jaspreet Singh Chhabra, Shashikant Mishra, S.B. Sudharsan, Arvind P. Ganpule, Ravindra B. Sabnis, Mahesh R. Desai

**Affiliations:** Department of Urology, Muljibhai Patel Urological Hospital, Nadiad, Gujarat, India.

## Abstract

Transitional-cell carcinoma (TCC) of the upper tract in a case of circumcaval ureter (CCU) is a rare entity. Laparoscopic transperitoneal nephroureterectomy in such case represents a unique challenge in the era of minimally invasive surgery. We report a case of complete transperitoneal laparoscopic nephroureterectomy with bladder cuff excision done for TCC in a case of CCU. This case report describes the first point of technique of the procedure done for this rare entity. A 38-year-old male patient underwent the procedure for high-grade TCC of right lower calix. The essential tenets of the procedure included performance of the technique in a manner contrary to the conventional nephroureterectomy. The case report describes the procedure in the following steps: management of lower ureter and bladder cuff followed upper tract procedure after transposition of bladder cuff posterior to inferior vena cava. The procedure was accomplished utilizing four ports and a 6 cm Pfannenstiel incision with operative time of 220 minutes and blood loss of 50 mL.

## Case History, Physical Examination, and Diagnosis

**FIG. 1.**
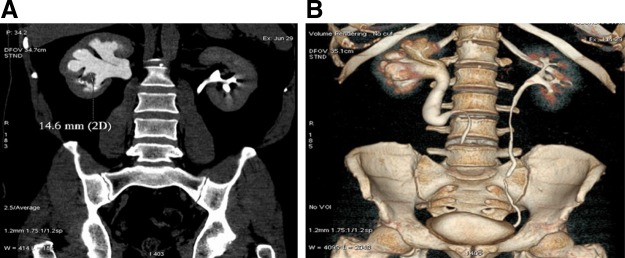
Imaging. **(A)** 18 × 14 mm enhancing irregularly marginated soft tissue mass lesion involving right lower calix. **(B)** Retrocaval right ureter.

A 38-year-old male with a body mass index of 28 kg/m^2^ and a heavy smoker presented with a history of intermittent gross painless hematuria with clots and occasional right flank pain of 1-year duration. Clinical examination was unremarkable. Investigations revealed a positive urine cytology. CT urography showed a filling defect and space occupying lesion in the right lower calix with a circumcaval course of the ureter (Fig. 1). The patient was planned for retrograde pyelography (RGP) and diagnostic flexible ureteroscopy. RGP confirmed a retrocaval ureter with a filling defect in the lower calix. Due to a tortuous circumcaval course, the flexible ureteroscope could not be negotiated. He subsequently underwent right percutaneous nephroscopy that confirmed the presence of papillary growth in the lower calix. Biopsy of the lesion revealed a high-grade transitional-cell carcinoma (TCC). The patient was counseled regarding the available treatment options and an informed consent for laparoscopic nephroureterectomy was obtained.

## Intervention

The authors surveyed the literature regarding the same and found no previous published technique of complete performance of the procedure in such a scenario by minimally invasive means. The literature confirms a published case report of retroperitoneoscopic nephroureterectomy; however, the details regarding the complete laparoscopic bladder cuff excision with a single *in toto* specimen were not available. To have a complete single specimen, we decided to perform this procedure tackling the lower tract first. This would enable easy transposition of the relatively smaller bladder cuff and lower ureter, winding round in a reverse manner to maintain a conventional course. The case report describes the procedure in two steps. Port position was planned in such a way that step 1 and 2 could be completed with a total of four ports. After induction of general anesthesia and per urethral catheterization, patient positioning was done to proceed for the first step of the procedure.

### Step 1 (bladder cuff and lower ureter)

**FIG. 2.**
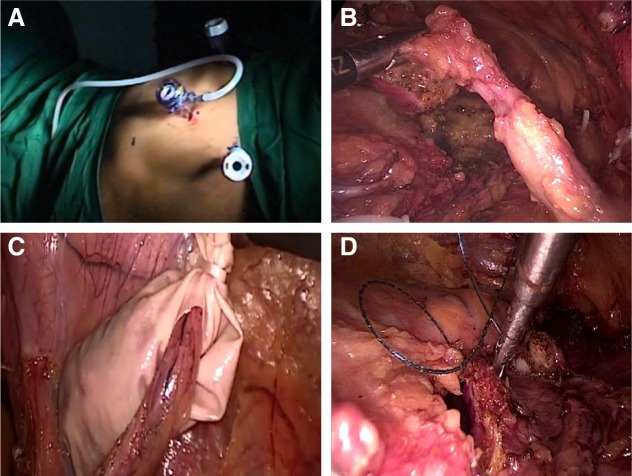
Step 1 (lower tract). **(A)** Port positioning in steep Trendelenburg position. **(B)** Lower ureter with bladder cuff. **(C)** Cuff placed in customized hand glove extraction bag. **(D)** Intracorporeal suture closure of cystotomy defect.

The patient was placed in a steep Trendelenburg position (Fig. 2A). Camera port (12 mm Excel™) was placed 3 cm above the umbilicus. Right hand (12 mm Excel) dissection port and left hand (5 mm Excel™) retraction port were placed at the midpoint of the anterior superior iliac spine and umbilicus on right and left sides, respectively. The procedure was started with mobilization of the right lower ureter after incising the peritoneal covering at the level of pelvic brim and dissecting the same till the vesicoureteric junction (VUJ). This was facilitated by dissection and ligature clipping of vas deferens, obliterated umbilical artery, superior vesical artery, and mobilization of bladder by releasing the peritoneal attachments. Incision was made at the VUJ and bladder cuff excised (Fig. 2B) under direct vision with a monopolar hook, avoiding injury to the contralateral ureteral orifice. The released bladder cuff was immediately placed in a specimen delivery bag made out of customized finger glove (Fig. 2C). The bladder was closed with intracorporeal single-layer continuous suturing using barbed V-Loc™ 2-0 sutures (Fig. 2D). Integrity of the bladder closure was then checked with saline instillation through Foleys catheter. The ports were taken out to mark the end of step 1 procedure. The left lower 5 mm port site was closed, while the umbilical and right hand port site were kept open to be utilized again in step 2.

### Step 2 (circum mobilization of the ureter along the inferior vena cava and nephrectomy)

**FIG. 3.**
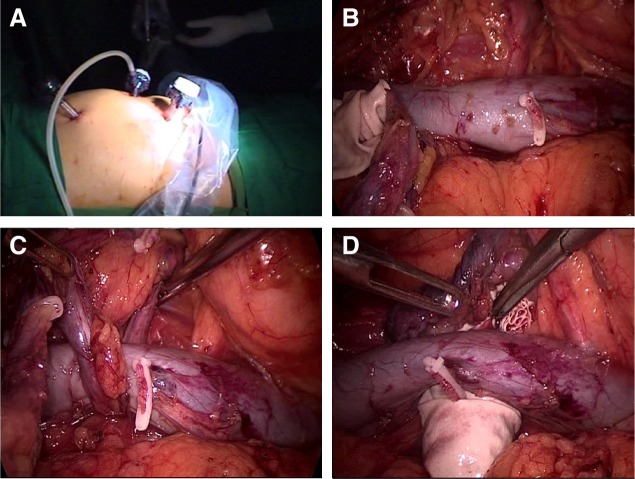
Step 2 (upper tract). **(A)** Port positioning in 90° flank position. **(B)** Dissection of inferior vena cava (IVC). **(C)** Mobilization of IVC using ureter as sling. **(D)** Transposition of bladder cuff and lower ureter posterior to IVC.

**FIG. 4.**
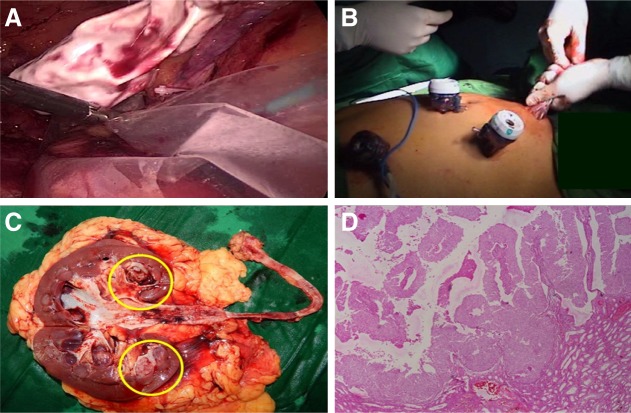
Retrieval and pathology. **(A)** Bagging of specimen. **(B)** Retrieval through Pfannenstiel incision. **(C)** Gross specimen. *Yellow* labels demonstrate lesion in lower calyx. **(D)** Histopathology image.

Undraping and change of patient position to 90° left flank position were done (Fig. 3A). After proper draping of the patient, the camera and left hand port (the step 1 right hand port) were placed, while an additional right hand port (12 mm Excel) was placed at the right subcostal location. The procedure was started by mobilizing the right colon after incising along the white line of Toldt. The inferior vena cava (IVC) was dissected completely both proximal and distal to the retrocaval course of the ureter (Fig. 3B). The posterior dissection of IVC was aided by utilizing the ureter itself as a sling (Fig. 3C). This maneuver helped in clipping a small lumbar vein tributary as well and ensuring an adequate space for the transposition of the bladder cuff–lower ureter complex behind the IVC (Fig. 3D), so that a conventional and more familiar orientation is attained. Completion nephrectomy was then performed in a standard manner. The complete nephroureterectomy specimen was placed in another specimen retrieval bag (Fig. 4A) and was retrieved through a 6 cm muscle splitting Pfannenstiel incision (Fig. 4B). Check laparoscopy was performed to ensure hemostasis and a drain was placed through the right lower port and the remaining ports were closed.

The total operative time was 220 minutes with a blood loss of about 50 mL. The patient had a smooth postoperative recovery with an analgesic requirement of 250 mg of tramadol. The drain was removed on the second postoperative day and the patient was discharged on the third postoperative day with the Foley catheter *in situ*. The latter was removed on the seventh postoperative day, during patient's outpatient department visit. The pathology report revealed a 1.5 × 1.5 cm lower caliceal polypoidal growth (Fig. 4C), suggestive of high-grade urothelial carcinoma (Fig. 4D). Cystoscopy performed at the end of 3 months showed no evidence of recurrence in the bladder, and a CT scan at the end of a 1-year follow-up period was normal.

## Discussion

To our knowledge, this is the first clinical case report suggesting the feasibility and efficacy of transperitoneal complete laparoscopic nephroureterectomy in a rare case of circumcaval ureter (CCU) with TCC. The case rarity can be appreciated by the fact that ever since the first report by Clements and colleagues,^1^ there have been only 11 cases reported so far.^2^ The plausible hypothesis ascribed to tumorigenesis in such a condition is the urine stasis occurring in a dilated collecting system of a CCU.^3^ With the advent of minimally invasive surgery (MIS), there are unique challenges that present to the operating surgeon; the foremost among them being the questionable wisdom of doing a traditional nephroureterectomy. It is not possible to remove the intact specimen without dismembering, since the large bulk of kidney is practically impossible to transpose. Therefore, it makes sense to do excisional surgery of lower tract first, before handling the kidney. Even for a latter circumstance, the second challenge is the transposition of lower ureter. The case reports described so far have not described the point of technique through which MIS can accomplish the task. The advent of MIS and application of laparoscopic or robotic surgery for treating such cases remain challenging. Our case report suggests the point of technique through which complete laparoscopic transperitoneal nephroureterectomy can be done and the entire procedure can be completed with four ports, which appears to be the minimum number required for accomplishing the procedure. By doing so, all the advantages of MIS were maintained along with oncologic efficacy. However, the main limiting factor appears to be the high degree of laparoscopic expertise required. The procedure was performed by a highly skilled laparoscopic surgeon and replicability of the procedure remains an addressing issue. The procedure is efficacious and is advisable to the operating surgeons if such a rare case is diagnosed in clinical practice.

## Abbreviations Used

CCU: circumcaval ureter
CT: computed tomography
IVC: inferior vena cava
MIS: minimally invasive surgery
RGP: retrograde pyelography
TCC: transitional-cell carcinoma
VUJ: vesicoureteric junction
